# The Role of Abnormal Methylation of Wnt5a Gene Promoter Regions in Human Epithelial Ovarian Cancer: A Clinical and Experimental Study

**DOI:** 10.1155/2018/6567081

**Published:** 2018-07-16

**Authors:** Ping Jin, Yi Song, Guiyuan Yu

**Affiliations:** ^1^The Shenzhen Maternity & Child Healthcare Hospital Affiliated to Southern Medical University, ShenZhen 518028, China; ^2^The First Clinical Medical College of Lanzhou University, Lanzhou, China

## Abstract

**Objective:**

In the current study, the role of abnormal methylation of Wnt5a gene promoter regions in human epithelial ovarian cancer was investigated.

**Methods:**

Wnt5a expressions were examined by immunohistochemistry in epithelial ovarian tissues (30 normal and 79 human EOC tissues). SKOV3 cells were treated with different concentrations of 5-Aza-CdR (0.5, 5, and 50 *μ*mol/L). The methylation status of the Wnt5a promoter was analyzed using a methylation-specific polymerase chain reaction (MSP), and the expression level of Wnt5a mRNA was detected using quantitative real-time polymerase chain reaction (qRT-PCR). Cell proliferation was measured by MTT assay, and apoptosis was analyzed using flow cytometry.

**Results:**

(1) Compared with normal tissues, Wnt5a expressions were reduced or lost in EOC (*P* < 0.05). Wnt5a expression had a close relationship with histological grade, FIGO stage, and lymph node metastasis (*P* = 0.005, *P* = 0.022, and *P* = 0.037, resp.). (2) Wnt5a abnormal methylation status existed in ovarian cancer tissues and was higher than that of normal ovarian tissue (*P* < 0.01). (3) Before treatment with 5-Aza-CdR, the promoter of the Wnt5a gene was methylated in SKOV3 cells; accordingly, Wnt5a mRNA levels were low to absent in SKOV3 cells. (4) Following 5-Aza-CdR treatment, MSP analysis revealed complete demethylation of the Wnt5a promoter in the SKOV3 cell line, particularly at 5 *μ*mol/L 5-Aza-CdR. Wnt5a expression increased in SKOV3 cells following treatment with a demethylating agent (*P* ≤ 0.001). (5) The growth rate of the cells was inhibited in a dose-dependent manner by treatment with 5-Aza-CdR. (6) The cell apoptosis rate increased gradually after treatment with 0.5, 5, and 50 *μ*mol/L 5-Aza-CdR. The apoptosis rate exists in a dose-dependent relationship with 5-Aza-CdR concentration (*F* = 779.73, *P* < 0.01).

**Conclusions:**

Wnt5a gene region promoter aberrant methylation existed in epithelial ovarian cancer, and abnormal methylation of Wnt5a gene promoter regions may be a new target for the treatment of epithelial ovarian cancer.

## 1. Introduction

The characteristics of ovarian cancer include a difficult early diagnosis, rapid development, and high mortality. More than 70% of ovarian cancer patients are diagnosed at an advanced stage, and the five-year survival rate is only 20%. As a result, ovarian cancer is the most lethal malignant tumor of all female reproductive system tumors; epithelial ovarian cancer (EOC) is the most common type and accounts for approximately 60%–90% of all ovarian cancers [1]. As a result of in-depth studies in recent years, abnormalities of epigenetic modifications have been found to be one of the important reasons for tumor formation, are involved in the occurrence and development process of tumors, and are closely related to some pathologic types and prognosis. Epigenetic modification includes DNA methylation, microRNA regulation abnormalities, and histone acetylation. Among the modifications, DNA methylation is one of the most important methods of epigenetic regulation, which can cause changes to chromatin structure, DNA conformation, DNA stability, DNA-protein interaction, and gene expression. Abnormal promoter methylation is the molecular basis of genomic instability, and abnormal gene expression of the methylation status of tumor-related genes is an early sensitive indicator of tumor development [2, 3].

Wnt signals are involved in adult cell proliferation, differentiation, and apoptosis. The signal transduction pathways include the classical Wnt pathway, nonclassical Wnt/JNK pathway, and Wnt/Ca^2+^ pathway; abnormal signal transduction pathways lead to tumor formation [4]. The Wnt5a gene, an important member of the Wnt family, is located on chromosome 3p, 14.2 p21.1. Wnt5a was firstly discovered by Clark et al., a molecular biologist at the University of Thomas Jefferson. The Wnt5a gene is composed of 1172 adenines, 884 cytosines, 946 guanines, and 1172 thymines. The gene contains 5 exons, and its terminal exon encodes the large 3′ end of the untranslated region. The promoter region is located in a region that is rich in glycerol phosphate choline and comprises many cis-acting elements [5, 6]. The 631 base pair Wnt5a gene initiation region contains strong promoter activity [7]. Wnt5a is increased, decreased, or deleted in different tumors and, as a result, plays different roles in tumors [8]. DNA methylation is a reversible epigenetic modification. The DNA methyltransferase inhibitor 5-aza-2′-deoxycytidine (5-Aza-CdR) inhibits the methylase enzyme DNMT, reverses hypermethylation of the promoter region to enable reexpression of tumor-associated genes, and inhibits tumor cell growth. In conclusion, the use of demethylating agents for cancer has become prominent in recent years [9].

The objective of this study was to understand the relationship between Wnt5a promoter methylation and epithelial ovarian cancer, observe the methylation status of the promoter region of the *Wnt5a* gene, explore the change in transcriptional expression and cell biological characteristics when treating with the demethylating agent 5-Aza-CdR, and finally provide a mechanistic basis to support the use of demethylating agents in the treatment of epithelial ovarian cancer.

## 2. Materials and Methods

### 2.1. Patients and Tissue Samples

Ninety-nine patients in the First Affiliated Hospital of Lanzhou University, China, from January 2009 to November 2013 were recruited including 79 patients of EOC and 30 cases of normal ovarian tissues The median age was 48 years (range from 27 to 73 years old). The experimental group comprised 79 untreated patients at different clinical stages: stage I (*n* = 15), stage II (*n* = 21), stage III (*n* = 29), and stage IV (*n* = 14). All the EOC patients were confirmed by histopathology including 50 serous adenocarcinomas, 23 mucinous adenocarcinomas, 4 endometrioid carcinomas, 1 Brenner's disease, and 1 clear-cell carcinoma. Their histological grades were G1 (*n* = 13), G2 (*n* = 29), and G3 (*n* = 37). Diagnosis was confirmed or excluded based on standard morphologic, cytochemical, and immunophenotypic criteria. Informed consent was obtained from all the participants.

### 2.2. Immunohistochemistry

Immunostaining was performed with paraffin-embedded sections that were cut at 4 *μ*m and mounted onto glass slides. Tissue sections were deparaffinized with xylene and rehydrated through graded alcohol. Then, the slides were incubated in 3% H_2_O_2_ for 10 min at room temperature so as to blocking endogenous peroxidase activity. Thereafter, antigen retrieval was performed in the citric acid repair solution for 5 seconds, 1700°C, then stewed 5 minutes, and finally rinsed with PBS. Finally, the sections were incubated with primary antibody to Wnt5a (1 : 500 dilutions, ab86720; Abcam, UK) overnight at 4°C. The next day, slides were stained with DAB and then counterstained applying hematoxylin. Wnt5a staining was shown in both the cytoplasm and the cytomembrane. The evaluation criterion of Wnt5a expression consists of both the staining intensity and the percentage of positive tumor cells. The former was graded as 0 to 3 (0: negative, 1: weak, 2: medium, and 3: strong). The latter was scored based on the percentage of the positive staining area in the total tumor area as follows: 0 (<1%), 1 (≤25%), 2 (26%–50%), 3 (51%–75%), and 4 (>75%). These two scores were summed up as final staining scores (0–7). The staining score of 0–2 was defined as low expression, 3-4 was called moderate expression, and 5–7 represented high expression. Thyroid cancer tissue was assigned as a positive control. On the contrary, the negative control was incubated with phosphate-buffered saline (PBS) alone instead of primary antibody.

### 2.3. Cell Culture and Drug Treatment

The SKOV3 ovarian cancer cell line was obtained from the Basic Medical Centre of Lanzhou University. The cells were maintained in RPMI 1640 supplemented with 10% FBS and 50 *μ*g/mL gentamicin in a humidified 5% CO_2_ atmosphere at 37°C. The SKOV3 tumor cells (2 × 10^5^ cells) were cultured for three days in the presence of various concentrations (0.5 *μ*mol/L, 5 *μ*mol/L, and 50 *μ*mol/L) of 5-aza-2′-deoxycytidine (5-Aza-CdR; Sigma, St. Louis, MO, USA). The cells were then cultured for an additional seven days in fresh culture medium without drugs to eliminate the influence of drug cytotoxicity. The control group was cultured in the absence of any agent.

### 2.4. Bisulphite Modification and Methylation-Specific PCR (MSP)

Bisulphite modification of genomic DNA was evaluated using the CpG genome DNA Methylation-Gold™ Kit (Zymo Research, Irvine, CA, USA). The quality and integrity of the DNA were determined by the A260/280 ratio. The primer sequences for MSP amplification were Wnt5a-MD (5′-GTATTTTTCGGAGAAAAAGTTATGC-3′) and Wnt5a-MR (5′-ACAACCGCGAATTAATATAAACG-3′) for the methylated reaction and Wnt5a-UD (5′-GGTATTTTTTGGAGAAAAAGTTATGTG-3′) and Wnt5a-UR (5′-CTACAACCACAAATTAATATAAACATC-3′) for the unmethylated reaction [10]. “Hot start” PCR was performed for 35 cycles, consisting of denaturation at 95°C for 1 min, annealing at 60°C for 1 min, and extension at 72°C for 1 min, followed by a final 7 min extension for both primer sets. The reaction products were separated by electrophoresis on 2% agarose gels. The DNA from blood marrow mononuclear cells treated with SssI methyltransferase (New England Biolabs, Ipswich, MA, USA) was used as a positive control for methylation. The results were confirmed by repeating the MSP assays following an independently performed bisulphite treatment.

### 2.5. Quantitative Real-Time Polymerase Chain Reaction (qRT-PCR)

Total RNA was isolated from the SKOV3 ovarian cell line using TRIzol (Shanghai Sangon Biological Engineering Technology & Services Co., China). All RNA samples were treated with TURBO DNase enzyme (TURBO DNA free kit; Ambion Inc., Applied Biosystems) to remove any contaminating DNA. Quantitative RT-PCR was performed using Takara Real-Time PCR System (Tokyo, Japan). The first-strand cDNA sample was then amplified using previously published primer sets for Wnt5a (5′-GTGCAATGTCTTCCAAGTTCTTC-3′ (top strand) and 5′-GGCACAGTTTCTTCTGTCCTTG-3′ (bottom strand)) [10] and *β*-actin (5′-GCATGGGTCAGAAGGATTCTT-3′ (top strand) and 5′-TCGTCCCAGTTGGTGACGAT-3′ (bottom strand)).

### 2.6. MTT Assay

Cells from the 5-Aza-CdR-treated or control groups were seeded in a 96-well plate at a density of 3 × 10^3^ cells/well and incubated for 7 d. Wells containing culture medium but with no cells were used as blanks. Cells were incubated with 20 *μ*L of 0.5 mg/mL MTT (Sigma) for 4 h at 37°C. After removing the medium, formazan crystals were resolubilized with 150 *μ*L of dimethyl sulfoxide (Sigma) for 10 min at 37°C. Absorbance at 570 nm was measured with a microplate reader. All test conditions were evaluated in triplicate wells on the same plates, and each experiment was repeated three times.

### 2.7. Annexin V and PI Double Staining of SKOV3 Apoptosis

SKOV3 cells from the 5-Aza-CdR-treated or control groups were resuspended at 6 × 10^5^ cells/mL, and 1 mL of this suspension was seeded into cell culture flasks. The culture medium was removed once cells adhered to the surface, and then 5-Aza-CdR was added to a final concentration of 0.5, 5, or 50 *μ*mol/L in 10 mL of medium. For the control group, an equivalent volume of culture medium without drugs was added. The cells were incubated for 48 h, collected, and resuspended in 0.5 mL of PBS, and then annexin V and PI were added. Cells were incubated in the dark for 10 min at room temperature and analyzed by flow cytometry.

### 2.8. Statistical Analysis

Quantitative data are reported as the mean ± standard deviation (SD). Pearson's *χ*^2^ test was used to analyze the relationships between Wnt5a immunohistochemical staining and clinicopathologic variables. Contingency table analysis and Pearson's *χ*^2^ test were applied to compare the Wnt5a methylation status among cases and between various clinicopathologic variables. The difference in *Wnt5a* mRNA expression before and after 5-Aza-CdR treatment was analyzed by a paired sample *t*-test. The percentage of apoptosis was evaluated by the LSD test. All statistical tests were two-sided and were performed at the 5% level of significance using the SPSS19.0 software package; *P* < 0.05 was considered significant.

## 3. Results

### 3.1. Wnt5a Expression by Immunohistochemistry in Normal Ovary Tissues and EOC

**Figure 1:**
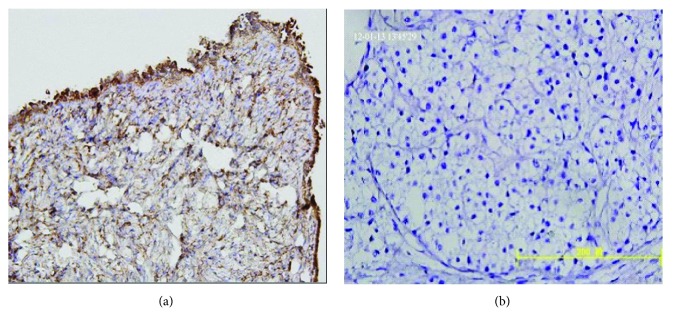
Expression of Wnt5a in normal ovarian tissue and ovarian cancer. Normal ovaries with immunostaining of Wnt5a in the cytoplasm of tumor cells. Staining index (SI = 7) in epithelial ovarian tissues. Immunolabeling in the cytoplasm of fibroblasts on the (a) (40x). Ovarian cancer was clear-cell cancer, in the cytoplasm of tumor cells (40x; SI = 0).

Staining of Wnt5a was presented mainly in the cytoplasm. As shown in Figure 1, Wnt5a expression was significantly higher in normal ovaries (21/30) than in epithelial ovarian carcinomas (35/79) (*P* = 0.017; *χ*^2^ = 5.747).

### 3.2. Methylation of Wnt5a in Human Epithelial Ovarian Carcinoma Tissues

**Figure 2:**
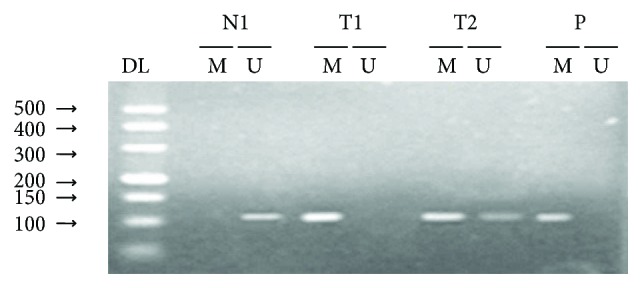
Methylation status of Wnt5a in normal ovarian tissues and epithelial ovarian carcinomas. N: normal ovarian tissues; T: epithelial ovarian carcinomas; P: positive control; DL500: DNA marker; M: methylation; U: unmethylated.

MSP was used for the analysis of the Wnt5a methylation status (Figure 2). 31 of 79 (39.24%) methylation were tested in EOC, while only 3 of 30 (10%) was found in normal ovarian tissues. The difference was statistically significant (*P* < 0.01).

### 3.3. The Relationship between Wnt5a Expression and Methylation with Clinicopathological Features of EOC

From Table 1, Wnt5a expression was related to the histological grade, FIGO stage, and lymph node metastasis (*P* = 0.005; *P* = 0.006; and *P* = 0.037, resp.). However, no significant difference was observed in other factors such as age and histological type.

For the part of methylation, statistical analysis showed no significant correlation between promoter region hypermethylation and age, histological type, or WHO grades. On the contrary, we observed an obvious relation between promoter region hypermethylation with FIGO stages and lymphatic metastasis (*P* = 0.018 and *P* = 0.024, resp.). The methylation was discovered in 9 of 36 (25%) FIGO stage I~II and in 22 of 43 (51.16%) FIGO stage III~IV; Wnt5a was methylated in 21 of 41 (51.22%) carcinomas with lymph node metastasis, while 10 of 28 (26.32%) tumors without lymph node metastasis.

### 3.4. Methylation of Wnt5a in the Human Epithelial Ovarian Cancer Cell Line SKOV3

**Figure 3:**
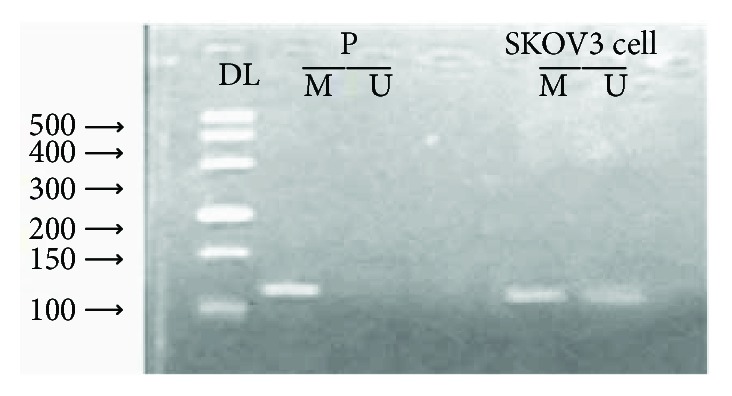
Methylation of Wnt5a in the human epithelial ovarian cancer cell line SKOV3. P: positive control; DL500: DNA marker; M: methylation; U: unmethylated.

The methylation status of *Wnt5a* in the human epithelial ovarian cancer cell line SKOV3 was determined by MSP analysis (Figure 3). The band corresponding to the methylated *Wnt5a* gene is indicated (107 bp). The primers recognizing the unmethylated gene were able to amplify a band of weaker intensity, suggesting that *Wnt5a* is partially methylated in this cell line.

### 3.5. 5-Aza-CdR Treatment Induces Demethylation of the Wnt5a Promoter and Increases mRNA Expression in the SKOV3 Cell Line

**Figure 4:**
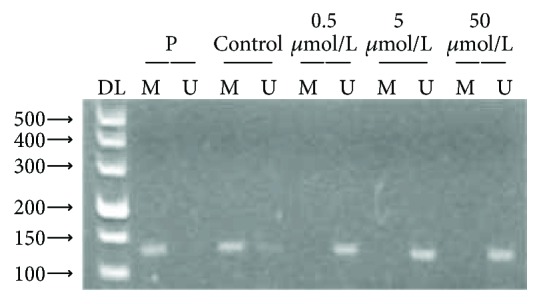
Demethylation of Wnt5a by different concentrations of 5-AzA-CdR in the SKOV3 cell line. DL500: DNA marker; P: positive control M: methylation; U: unmethylated.

SKOV3 cells were treated with different concentrations of the demethylating agent 5-Aza-CdR to determine whether methylation of the *Wnt5a* promoter is the basis for the low level of expression. The MSP analysis revealed complete demethylation of the *Wnt5a* promoter in the SKOV3 cell line following 5-Aza-CdR treatment (Figure 4).

**Figure 5:**
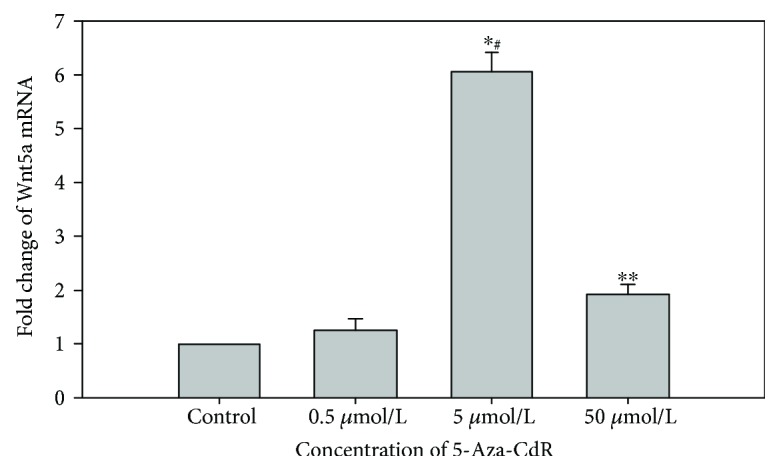
Expression of Wnt5a gene mRNA with different concentrations of 5-Aza-CdR in the SKOV3 cell line. ^#^Compared with the control group, (^∗^*P* < 0.01, ^∗∗^different concentrations of 5-Aza-CdR).

The results of mRNA expression after 5-Aza-CdR treatment are shown in Figure 5, which represented that *Wnt5a* expression was increased in SKOV3 cells following treatment with the demethylating agent (*P* ≤ 0.001). These data indicated that promoter methylation was closely correlated with low levels of Wnt5a expression in the SKOV3 cell line.

### 3.6. The Impact of 5-Aza-CdR Treatment on the Growth Rate of SKOV3 Cells

**Figure 6:**
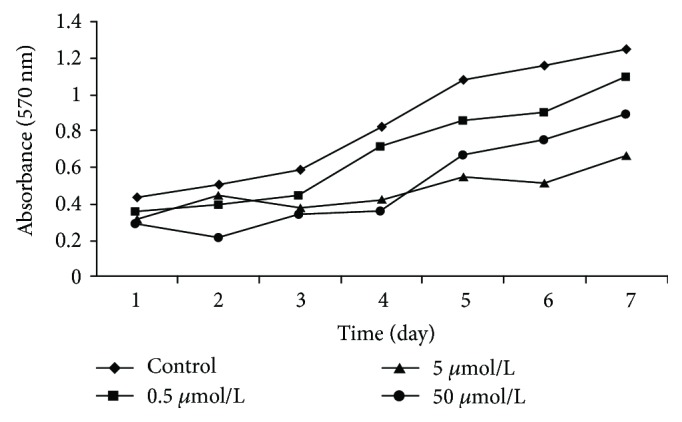
SKOV3 cell proliferation in a time- and dose-dependent manner.

The growth rate of the cells was inhibited in a dose-dependent manner by treatment with 5-Aza-CdR. In the first 96 h, we found that SKOV3 cell growth was inhibited in different concentrations of 5-Aza-CdR. And the 50 *μ*mol/L 5-Aza-CdR has the higher effects than other groups. But after 96 h, all drugs' group changed subtly, but it is strange that the inhibition of 50 *μ*mol/L 5-Aza-CdR was attenuated (Figure 6).

### 3.7. The Influence of 5-Aza-CdR on the SKOV3 Cell Apoptosis Rate

**Figure 7:**
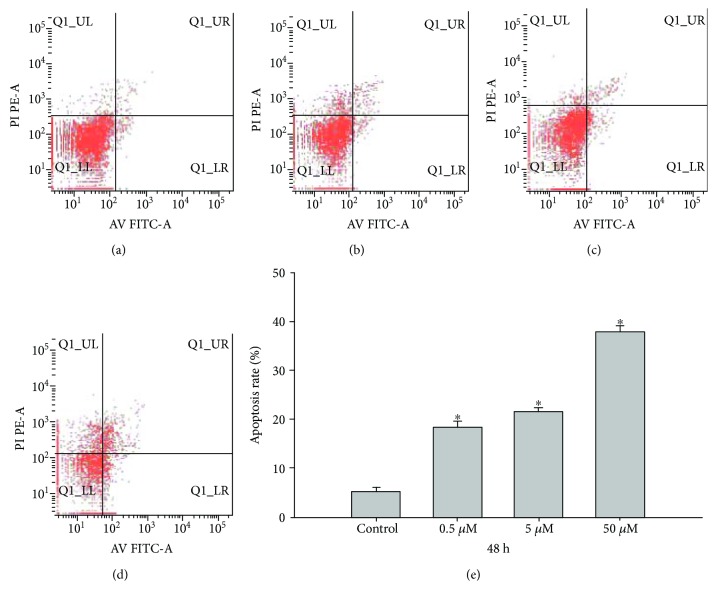
The rate of apoptosis and 5-Aza-CdR concentration show a dose-dependent relationship (^∗^*P* < 0.01). (a) Control. (b) 0.5 *μ*mol/L dose group. (c) 5 *μ*mol/L dose group. (d) 50 *μ*mol/L dose group. (e) The statistics of apoptosis rate in each group.

By flow cytometry analysis, the cell apoptosis rate increased gradually after treatment with 0.5, 5, and 50 *μ*mol/L 5-Aza-CdR; the apoptosis rates were 4.24% ± 0.79%, 9.20% ± 0.26%, and 20.48% ± 1.89%, respectively, compared with the control group (1.89% ± 0.48%) (*P* < 0.01). The apoptosis rate and 5-Aza-CdR concentration show a dose-dependent relationship (*F* = 779.73, *P* < 0.01) (Figure 7).

## 4. Discussion

DNA methylation refers to the catalytic action of DNA methyltransferase (DNMT1, DNMT3A, and DNMT3B), which can covalently combine the S-adenosylmethionine (SAM) to the CpG dinucleotide 5-carbon position of cytosine to result in the formation of 5-methylcytosine using s-adenosylmethionine (SAM) as a methyl donor [11]. CpG dinucleotides are the major site of DNA methylation, which is nonrandomly distributed throughout the genome. Typically, DNA sequences with a CpG content that is more than 50% and length that is greater than 200 bp are called CpG islands (CpG islands (CGIs)) [12]. In mammals, DNA methylation occurs mostly in the CpG island cytosine, playing an important role in the regulation of gene expression. A considerable part of methylation occurs outside the CpG island region (non-CpG site), but the role of this methylation is not clear [13, 14]. CpG islands are mainly located in the promoter region of the gene, typically in a nonmethylated form, but when tumors occur, in particular, hypermethylation occurs in the CpG islands of tumor suppressor genes (TSG) [15, 16].

The *Wnt5a* gene is an important member of the Wnt family. Researchers have found that *Wnt5a* can be involved in the occurrence and development of tumors by various manners. Wnt5a can affect cell migration, invasion, and angiogenesis in tissues; however, it is also conducive to tissue repair and maintenance of the steady state of tissues [16]. Some studies demonstrated that *Wnt5a* functions through the Wnt/Ca^2+^ pathway in some tumors. *Wnt5a* can not only activate but also inhibit the canonical Wnt/*β*-catenin pathway, depending on what type of receptor *Wnt5a* combines with. When *Wnt5a* binds to Ror2, the canonical Wnt/*β*-catenin pathway is being inhibited by the Wnt/Ca^2+^ pathway. However, when *Wnt5a* binds to Frizzled and LRP, the opposite effect occurs. Thus, whether *Wnt5a* suppresses or leads to cancer formation depends on the cell surface receptor it binds [17–19]. In addition, expression of the *Wnt5a* gene may be increased, decreased, or absent in tumors. *Wnt5a* methylation also differs among tumors, and the mechanism of *Wnt5a* methylation in tumor occurrence and development differs. As a result, *Wnt5a* plays dual roles. In some tumors, such as melanoma, gastric cancer, osteosarcoma, prostate cancer, nonsmall cell lung cancer, nasopharyngeal carcinoma, and pancreatic cancer, *Wnt5a* is overexpressed and plays the role of an oncogene [20–24]. In other tumors, such as colorectal cancer, esophageal squamous cell carcinoma, thyroid carcinoma, breast cancer, and white blood cell disease, *Wnt5a* shows low expression or loss of expression and plays the role of a tumor suppressor gene [25–28].

In our study, we described that Wnt5a expression was significantly higher in normal ovaries than in ovarian carcinomas with a proportion of 70% to 44.3%. In addition, Wnt5a expression was related to the histological grade, FIGO stage, and lymph node metastasis but was not associated with age and histological type (Figure 1, Table 1). Bitler et al. [29] also found that *Wnt5a* gene expression levels were significantly lower in EOC patients, and low levels of *Wnt5a* expression were significantly related to tumor staging and predicted shorter overall survival in patients when compared with normal ovarian surface epithelial cells or fallopian tube epithelial cells. Notably, increased *Wnt5a* expression significantly inhibited the proliferation of human EOC cells and ultimately played a role in promoting cell senescence in EOC. This is consistent with our experimental results, supporting the possible tumor suppressor gene characteristics of *Wnt5a* in ovarian cancer. However, some scholars have found that when compared to a benign epithelial neoplasia group and normal ovary group, the proportion of *Wnt5a*-positive women was significantly higher for the epithelial ovarian cancer group [30]. This result may not be consistent with the results of our study, but the results of our experiments have been demonstrated by both in vivo and in vitro experiments, and related research also found similar results. In addition, Bitler et al. [29] found that increased *Wnt5a* expression significantly inhibited the proliferation of human EOC cells. As a result, the differences in the conclusions of this study should be further studied and discussed to explain the reasons for these differences.

DNA methylation is widespread in epithelial ovarian tumors. Ho et al. [31] found that the methylation profiles of tumor suppressor genes (TSGs) were significantly higher in the ovarian cancer stromal progenitor cells (OCSPCs) than in ovarian cancer cells. OCSPCs and decreased TSG expression in the ovarian tumor microenvironment were able to promote tumorigenesis, which could be reversed by DNA demethylation. In the current study, the methylation status of Wnt5a in the human epithelial ovarian cancer was determined by MSP analysis (Figures 2 and 3). Our data show that Wnt5a promoter region abnormal methylation did exist in EOC and was closely associated with clinical progression of EOC (Table 1). In addition, a study found that CpG island methylation in ovarian cancer is related to the silencing of many genes, including *BRCA1*, *RASSF1A*, *LOT1* and *Hmlh1*; the silencing of these genes may be involved in the development of ovarian cancer [16]. The inactivation of tumor suppressor genes is related to hypermethylation, whereas genome-wide hypomethylation and local (CpG island) hypermethylation in tumors may be caused by disruptions of DNMT activity [32].

Our study further revealed complete demethylation of the *Wnt5a* promoter in the SKOV3 cell line following 5-Aza-CdR treatment (0.5, 5, and 50 *μ*mol/L) (Figure 4). The expression of *Wnt5a* mRNA was increased (Figure 5), particularly at 5 *μ*mol/L 5-Aza-CdR. These data indicated that the hypermethylation status of the promoter region of the *Wnt5a* gene in SKOV3 cells was reversed and that the *Wnt5a* mRNA was reexpressed. DNA methylation is a reversible epigenetic modification process that can be reversed by demethylation. 5-Aza-2-deoxycytidine (5-Aza-CdR) is now widely recognized as a demethylation agent. 5-Aza-CdR inhibits the methylase enzyme DNMT and stops cell DNA replication. Then, the methyl can not be transferred to the cytosine, thereby reducing the DNA methyl-accepting ability to reduce the degree of methylation in the progeny of the cells; thus, the tumor suppressor gene, whose promoter region is hypermethylated, finally reverses the biological activity of tumor cells [10]. 5-Aza-2′-deoxycytidine treatment not only reactivates genes but also decreases the overexpression of genes [33]. The difference in the two concentrations of 5-Aza-2-deoxycytidine (5-Aza-CdR) influences genome-wide methylation and induces changes in different sugar chains [34]. 5-Aza-CdR methylation inhibitors have been widely used in experimental studies of different tumors, such as colorectal cancer, breast cancer, thyroid cancer, and leukemia [35, 36].

In the current study, the growth rate of the tumor cells was inhibited by treatment with 5-Aza-CdR (Figure 6). A strange phenomenon in this result was that the inhibition of 50 *μ*mol/L 5-Aza-CdR was attenuated. Two reasons may explain this strange phenomenon; one is the widespread hypomethylation of the Wnt5a genome in all drugs' group. The other is that the drug of 5-Aza-CdR may have reached half-life and the effect starts to wane, which was supported by some researches [37]. The study suggested that 5-Aza-CdR can inhibit the proliferation of SKOV3 cells, and the inhibition was gradually increased with increasing drug concentration. This inhibition has been applied in many tumors. In breast cancer, DAC (5-aza-2′-deoxycytidine) treatment caused significant breast cancer stem cell (BCSC) differentiation. DAC reduced breast cancer cell survival and induced differentiation through reexpression of tumor suppressor genes [36].

In addition, our research showed that the cell apoptosis rate increased gradually after treatment with 0.5, 5, and 50 *μ*mol/L 5-Aza-CdR. The rate apoptosis and 5-Aza-CdR concentration share a dose-dependent relationship (Figure 7). 5-Aza-CdR efficiently induced cell cycle arrest at G0/G1 and apoptotic death in HXO-RB44 cells. An MSP analysis showed that unmethylated RASSF1A DNA increased and methylated RASSF1A decreased in a dose-dependent manner in a range of 0.5–5.0 *μ*mol/L 5-Aza-CdR. 5-Aza-CdR inhibits the growth of the HXO-RB44 RB cell line and induces apoptosis by demethylating the *RASSF1A* gene [38]. Several previous studies reported that 5-Aza-CdR exhibited cytotoxicity but the cells demonstrated cytotoxicity and that the effect was not hereditary [39]. In this experiment, we replaced 5-Aza-CdR with fresh medium for 7 days to avoid the toxic effects of 5-Aza-CdR. Therefore, the growth inhibition and apoptosis of cells in this experiment were a true reflection of 5-Aza-CdR demethylation.

In summary, Wnt5a gene region promoter aberrant methylation existed in epithelial ovarian cancer tissue, which is one of the important mechanisms of Wnt5a gene inactivation. 5-Aza-CdR treatment reversed the methylation status of the promoter and restored *Wnt5a* gene expression, which behaved as a tumor suppressor. Significantly, 5-Aza-CdR inhibited proliferation and induced apoptosis of SKOV3 cells due to the reversal of promoter hypermethylation of genes, including *Wnt5a*. Based on our results, *Wnt5a* demethylation may be a new target for the treatment of epithelial ovarian cancer.

## Figures and Tables

**Table 1:** Relationship between Wnt5a immunohistochemistry and methylation with clinicopathologic features of ovarian carcinoma.

Clinicopathologic parameters	*n*	Protein expression	*P*	Methylation positive	*P*
Age (years)					
≤48	34	14 (41.18)	>0.05	13 (38.24)	
>48	45	21 (46.47)		18 (40)	>0.05
WHO grades					
G1	13	10 (76.92)	0.005^∗^	5 (38.46)	
G2	29	13 (44.83)		10 (34.48)	>0.05
G3	37	12 (32.43)		16 (43.24)	
FIGO stages					
I~II	36	22 (61.11)	0.006	9 (25)	
III~IV	43	13 (30.23)		22 (51.16)	0.018
Histological types					
Serous adenocarcinoma	50	22 (44)	>0.05	19 (38)	
Mucinous adenocarcinoma	23	11 (47.83)		9 (39.13)	>0.05
Others	6	3 (50)		2 (33.33)	
Lymph node metastasis					
No	38	20 (52.63)	0.037	10 (26.32)	0.024
Yes	41	15 (36.59)		21 (51.22)	

^∗^The *P* value of G1 compared with that of G3.

## References

[1] Lengyel E. (2010). Ovarian cancer development and metastasis. *The American Journal of Pathology*.

[2] Robertson K. D. (2005). DNA methylation and human disease. *Nature Reviews Genetics*.

[3] Costa F. F. (2010). Epigenomics in cancer management. *Cancer Management and Research*.

[4] Yu J., Virshup D. M. (2014). Updating the Wnt pathways. *Bioscience Reports*.

[5] Kühl M. (2004). The WNT/calcium pathway: biochemical mediators, tools and future requirements. *Frontiers in Bioscience*.

[6] Clark C. C., Cohen I., Eichstetter I. (1993). Molecular cloning of the human proto-oncogene *Wnt*-5A and mapping of the gene (WNT5A) to chromosome 3p14-p21. *Genomics*.

[7] Prieve M. G., Moon R. T. (2003). Stromelysin-1 and mesothelin are differentially regulated by Wnt-5a and Wnt-1 in C57mg mouse mammary epithelial cells. *BMC Developmental Biology*.

[8] Chiba N., Furukawa K.-I., Takayama S. (2015). Decreased DNA methylation in the promoter region of the *WNT5A* and *GDNF* genes may promote the osteogenicity of mesenchymal stem cells from patients with ossified spinal ligaments. *Journal of Pharmacological Sciences*.

[9] Daskalakis M., Nguyen T. T., Nguyen C. (2002). Demethylation of a hypermethylated P15/INK4B gene in patients with myelodysplastic syndrome by 5–Aza–2′–deoxycytidine (decitabine) treatment. *Blood*.

[10] Roman-Gomez J., Jimenez-Velasco A., Cordeu L. (2007). *WNT5A*, a putative tumour suppressor of lymphoid malignancies, is inactivated by aberrant methylation in acute lymphoblastic leukaemia. *European Journal of Cancer*.

[11] Jeltsch A. (2002). Beyond Watson and Crick:DNA methylation and molecular enzymology of DNA methyltransferases. *ChemBioChem*.

[12] Smith Z. D., Meissner A. (2013). DNA methylation: roles in mammalian development. *Nature Reviews Genetics*.

[13] Law J. A., Jacobsen S. E. (2010). Establishing, maintaining and modifying DNA methylation patterns in plants and animals. *Nature Reviews Genetics*.

[14] Xie W., Barr C. L., Kim A. (2012). Base-resolution analyses of sequence and parent-of-origin dependent DNA methylation in the mouse genome. *Cell*.

[15] Long H. K., King H. W., Patient R. K., Odom D. T., Klose R. J. (2016). Protection of CpG islands from DNA methylation is DNA-encoded and evolutionarily conserved. *Nucleic Acids Research*.

[16] Poh W. J., Wee C. P. P., Gao Z. (2016). DNA methyltransferase activity assays: advances and challenges. *Theranostics*.

[17] Masckauchán T. N. H., Agalliu D., Vorontchikhina M. (2006). Wnt5a signaling induces proliferation and survival of endothelial cells in vitro and expression of MMP-1 and Tie-2. *Molecular Biology of the Cell*.

[18] Yuan Y., Niu C. C., Deng G. (2011). The Wnt5a/Ror2 noncanonical signaling pathway inhibits canonical Wnt signaling in K562 cells. *International Journal of Molecular Medicine*.

[19] Mikels A. J., Nusse R. (2006). Purified Wnt5a protein activates or inhibits *β*-catenin-TCF signaling depending on receptor context. *PLoS Biology*.

[20] Li J.-z., Zhang Y., Wen B., Li M., Wang Y.-j. (2015). Ability of *PITX2* methylation to predict survival in patients with prostate cancer. *OncoTargets and Therapy*.

[21] Hibi K., Sakata M., Yokomizi K. (2012). Methylation of the WNT5A gene is frequently detected in early gastric carcinoma. *Hepato-Gastroenterology*.

[22] Ekström E. J., Sherwood V., Andersson T. (2011). Methylation and loss of secreted frizzled-related protein 3 enhances melanoma cell migration and invasion. *PLoS One*.

[23] Vaidya H., Rumph C., Katula K. S. (2016). Inactivation of the *WNT5A* alternative promoter B is associated with DNA methylation and histone modification in osteosarcoma cell lines U2OS and SaOS-2. *PLoS One*.

[24] Bo H., Gao L., Chen Y., Zhang J., Zhu M. (2016). Upregulation of the expression of Wnt5a promotes the proliferation of pancreatic cancer cells in vitro and in a nude mouse model. *Molecular Medicine Reports*.

[25] Li J., Ying J., Fan Y. (2010). WNT5A antagonizes WNT/*β*-catenin signaling and is frequently silenced by promoter CpG methylation in esophageal squamous cell carcinoma. *Cancer Biology & Therapy*.

[26] Rawson J. B., Mrkonjic M., Daftary D. (2011). Promoter methylation of *Wnt5a* is associated with microsatellite instability and BRAF V600E mutation in two large populations of colorectal cancer patients. *British Journal of Cancer*.

[27] Hatırnaz Ng Ö., Fırtına S., Can İ. (2015). A possible role for WNT5A hypermethylation in pediatric acute lymphoblastic leukemia. *Turkish Journal of Hematology*.

[28] Trifa F., Karray-Chouayekh S., Jmal E. (2013). Loss of WIF-1 and Wnt5a expression is related to aggressiveness of sporadic breast cancer in Tunisian patients. *Tumour Biology*.

[29] Bitler B. G., Nicodemus J. P., Li H. (2011). Wnt5a suppresses epithelial ovarian cancer by promoting cellular senescence. *Cancer Research*.

[30] Badiglian Filho L., Oshima C. T., De Oliveira Lima F. (2009). Canonical and noncanonical Wnt pathway: a comparison among normal ovary, benign ovarian tumor and ovarian cancer. *Oncology Reports*.

[31] Ho C. M., Shih D. T., Hsiao C. C., Huang S. H., Chang S. F., Cheng W. F. (2015). Gene methylation of human ovarian carcinoma stromal progenitor cells promotes tumorigenesis. *Journal of Translational Medicine*.

[32] Ozdemir F., Altinisik J., Karateke A., Coksuer H., Buyru N. (2012). Methylation of tumor suppressor genes in ovarian cancer. *Experimental and Therapeutic Medicine*.

[33] Yang X., Han H., De Carvalho D. D., Lay F. D., Jones P. A., Liang G. (2014). Gene body methylation can alter gene expression and is a therapeutic target in cancer. *Cancer Cell*.

[34] Klasić M., Krištić J., Korać P. (2016). DNA hypomethylation upregulates expression of the *MGAT3* gene in HepG2 cells and leads to changes in *N*-glycosylation of secreted glycoproteins. *Scientific Reports*.

[35] Yang S., Wu B., Sun H. (2016). Interrupted E2F1-*miR-34c*-SCF negative feedback loop by hyper-methylation promotes colorectal cancer cell proliferation. *Bioscience Reports*.

[36] Phan N. L.-C., Van Trinh N., Van Pham P. (2016). Low concentrations of 5-aza-2′-deoxycytidine induce breast cancer stem cell differentiation by triggering tumor suppressor gene expression. *OncoTargets and Therapy*.

[37] Zhivkova Z. D., Mandova T., Doytchinova I. (2015). Quantitative structure – pharmacokinetics relationships analysis of basic drugs: volume of distribution. *Journal of Pharmacy & Pharmaceutical Sciences*.

[38] Liu R., Zhang X. H., Zhang K. (2014). 5-Aza-2′-deoxycytidine inhibits retinoblastoma cell by reactivating epigenetically silenced *RASSF1A* gene. *International Journal of Ophthalmology*.

[39] Wheeler J. M. D., Beck N. E., Kim H. C., Tomlinson I. P. M., McC. Mortensen N. J., Bodmer W. F. (1999). Mechanisms of inactivation of mismatch repair genes in human colorectal cancer cell lines: the predominant role of hMLH1. *Proceedings of the National Academy of Sciences of the United States of America*.

